# Antioxidant Strategies and Respiratory Disease of the Preterm Newborn: An Update

**DOI:** 10.1155/2014/721043

**Published:** 2014-04-07

**Authors:** Chiara Poggi, Carlo Dani

**Affiliations:** ^1^Neonatal Intensive Care Unit, Careggi University Hospital, 3 Largo Brambilla, 50141 Florence, Italy; ^2^Section of Neonatology, Department of Neurosciences, Psychology, Drug Research and Child Health, Careggi University Hospital, 3 Largo Brambilla, 50141 Florence, Italy

## Abstract

Preterm newborns are challenged by an excessive oxidative burden, as a result of several perinatal stimuli, as intrauterine infections, resuscitation, mechanical ventilation, and postnatal complications, in the presence of immature antioxidant capacities. “Oxygen radical disease of neonatology” comprises a wide range of conditions sharing a common pathway of pathogenesis and includes bronchopulmonary dysplasia (BPD) and other main complications of prematurity. Antioxidant strategies may be beneficial in the prevention and treatment of oxidative stress- (OS-) related lung disease of the preterm newborn. Endotracheal supplementation or lung-targeted overexpression of superoxide dismutase was proved to reduce lung damage in several models; however, the supplementation in preterm newborn failed to reduce the risk of BPD, although long-term respiratory outcomes were improved. Also melatonin administration to small cohorts of preterm newborns suggested beneficial effects on lung OS. The possibility to identify single nucleotide polymorphism affecting the risk of BPD may help to identify specific populations with particularly high risk of OS-related diseases and may pose the basis for individually targeted treatments. Finally, surfactant replacement may lead to local anti-inflammatory and antioxidant effects, thanks to specific enzymatic and nonenzymatic antioxidants naturally present in animal surfactants.

## 1. Introduction

Oxidative stress (OS) plays a pivotal role in the pathogenesis of several pathologic conditions of the preterm newborn, which are commonly referred to as “oxygen radical diseases of neonatology” [1]. In fact, the main complications of prematurity are located within the wide spectrum of OS-related injury, where bronchopulmonary dysplasia (BPD), retinopathy of prematurity, intraventricular hemorrhage, and periventricular leukomalacia are recognized as organ-specific manifestations [2, 3].

Preterm newborns are subjected to several events leading to increased reactive oxygen species (ROS) production, as hyperoxia, mechanical ventilation, and inflammatory and infective complications [2–5]. Resuscitation of preterm newborns with a low oxygen strategy providing FiO_2_ <0.3 significantly reduces the need for respiratory support and the incidence of BPD and OS markers [6] while a positive relation was demonstrated between cord levels of total hydroperoxides, advanced oxidation protein products, and non-protein-bound iron and the risk OS-related diseases of prematurity, including BPD [4]. As in the latest years resuscitation of term newborns with low FiO_2_ was associated with improved outcome [7] initial FiO_2_ of 0.21 is recommended at present by ILCOR guidelines [8] and stepwise titration of FiO_2_ basing on early SpO_2_ assessment in the delivery room is advised for preterm newborns [9–12]. Mechanical ventilation subsequently triggers inflammation pathways and remodeling processes in the developing lung [3, 13]. Therefore, very early activation of oxidative pathways in the preterm newborn may significantly affect long-term respiratory outcomes, indicating urgent need for specific counterbalance measures.

Antioxidant capacities are inadequate to the elevated oxidative burden in preterm newborns, both because of placental-fetal transfer interruption of antioxidant molecules and insufficient endogenous production [5, 14]. In fact, fetal levels of antioxidant enzymes (AOEs) as superoxide dismutase (SOD), catalase (CAT), glutathione peroxidase (GPx), and reductase (GRx) progressively increase during gestation, paralleling the maturation of surfactant system and preterm animal models fail to induce SOD and GPx in response to oxidative challenges [15, 16], in contrast to term newborns, who were demonstrated to induce AOEs in case of fetal distress [17] or resuscitation with high FiO_2_ [18].

Besides hyperoxic challenges, also hypoxia is a well-known activator of OS pathways in the newborn and ROS are believed to play a central role in the pathogenesis of hypoxic-ischemic encephalopathy (HIE) and cerebral palsy [19, 20]. Higher levels of OS markers were detected in the central nervous system of term newborns who suffered from HIE in comparison with nonasphyxiated controls, and a positive correlation was also established between the levels of OS markers and the severity of HIE [19]. Significant accumulation of ROS in the central nervous system was demonstrated in animal models of perinatal asphyxia followed by reoxygenation [21]. Moreover, iron release from erythrocytes and non-protein-bound iron resulted higher in asphyxiated newborns compared to normoxic controls [22] and similar results were obtained in isolated erythrocytes subjected to hypoxia-reoxygenation challenge [23, 24] suggesting a possible contribution of this aspect to ROS formation and OS damage perpetuation after hypoxic insult [25]. However, the role of hypoxic events in determining adverse respiratory outcomes in the preterm newborns has not been investigated so far.

Indeed, recent lines of evidence suggest that the administration of drugs exerting definite antioxidant and anti-inflammatory effects or inhibiting downstream enzymes of OS pathways is associated with improved outcomes in animal models of perinatal asphyxia or hypoxia-reoxygenation. Particularly, sodium hydrosulfide, doxycycline, and cyclosporine were demonstrated to improve cardiac function [26–28], while pyrrolidine dithiocarbamate and cyclosporine were proved to confer neuroprotection [29, 30].

## 2. Antioxidant Treatments: State of the Art

The present management of respiratory disease in the preterm newborn is far from optimal, as massive improvement in the treatment of the acute phase of RDS did not substantially impact on the incidence of BPD and also the management of patients with BPD relies on poor evidence [3, 31]. Several observations suggest beneficial effects of antioxidant therapies in the prevention of hyperoxia-induced lung injury, and because OS is well known to play a central role in the pathogenesis of BPD, it appears reasonable to consider the potentials of antioxidant strategies in the prevention and treatment of such conditions. However, antioxidant treatments cannot be at present recommended for such indications, as data from clinical trials are insufficient; therefore further researches on this topic are urgently needed in order to evaluate the clinical potential of antioxidants. The main results obtained with melatonin and AOEs supplementation or overexpression are reviewed.

### 2.1. Melatonin

Melatonin (MT) is a pleiotropic molecule secreted by the pineal gland as circadian rhythm transducer and exerts a complex of multiple antioxidant effects through direct and indirect actions on different pathways [32]. MT is a direct antioxidant as it presents scavenger properties against ROS, particularly hydroxyl radical, with subsequent formation of active metabolites which in turn show scavenging activity [33]. Moreover, MT upregulates the expression of AOEs, particularly GPx, GRx, SOD, and CAT [32] through the activation of different intracellular signaling pathways as those triggered by G-proteins, calcium-calmodulin complex, and NFkB [32]. In neonatal medicine MT is at present one of the most promising treatments for hypoxic-ischemic encephalopathy in addition to hypothermia [34].

In a small cohort of preterm newborns with RDS MT was proved to reduce the nitrite/nitrate ratio and circulating levels of oxidative and inflammatory markers which are typically overexpressed in lung tissues of BPD patients, as IL-6, IL-8, and TNF-alpha [35]. Moreover MT administration in 110 preterm newborns with RDS was associated with significantly lower ventilator parameters and lower circulating levels of IL-6, IL-8, and TNF-alpha in comparison with untreated controls [36]. Although these studies did not provide data on long-term respiratory outcomes, MT may be putatively expected to affect the incidence of BPD in preterm newborns, as inflammatory cytokines overexpression and lung recruitment of inflammatory cells are main steps in the pathologic pathway leading to tissue abnormalities typical of BPD [3]. Moreover, the beneficial effects of MT administration on inflammatory markers in RDS patients are concordant with the observation that MT also reduced circulating markers of lipid peroxidation in a small cohort of newborns diagnosed with sepsis [37]. Interestingly, significantly lower levels of lipid peroxidation markers were observed in the MT-treated group in comparison to the placebo group as early as 1 hour after the first administration [37], suggesting a very precocious activation of MT-induced antioxidant pathways, which would be of major importance in the setting of early postnatal prevention of OS-related complications.

Encouraging results were also obtained in the mouse model of BPD [38]. In fact, MT-treated animals showed reduced lung myeloperoxidase and nitrite/nitrate ratio and increased GPx, SOD, and CAT activity and a more preserved lung histopathology, suggesting a possible protective role of MT during the critical processes of alveolization and lung architecture development in case of unfavorable conditions, following preterm birth.

As very few data are available on the use of MT in preterm newborns, further researches are definitely needed in order to investigate the possible contribution of MT to the preventive strategies of OS and inflammation mediated lung injury. Promising preliminary results and the favorable safety profile along with high feasibility of administration pose the basis for future studies on MT in the prevention of BPD.

### 2.2. Antioxidant Enzymes Replacement

The rationale of AOEs replacement therapy in preterm newborn in order to prevent or attenuate adverse respiratory outcomes is based on data demonstrating that OS is a key activator of respiratory disease in the preterm newborn, who lack adequate levels of AOEs. Results of studies investigating treatment with AOEs in the respiratory disease of the preterm newborn are summarized in Table 1, while experiments on animal models are included in Table 2.

Several studies preliminarily proved that SOD is a crucial enzyme for a proper respiratory function as in animal and cellular models the absence of SOD3 activity induces severe lung injury during hyperoxia, while SOD2 overexpression in type II alveolar cells is associated with prolonged survival in hyperoxic environment [52, 53] and SOD3 administration in animal models was proved to reduce lung injury related to hyperoxia and mechanical ventilation [44–47]. Moreover, exogenously administered SOD was proved to be rapidly incorporated into different lung cell types after endotracheal administration in animal models [46], suggesting the possibility of a reliable route of administration into the lung.

Endotracheal administration of recombinant human SOD (rhSOD) has been proved to reduce lung injury in preterm newborns receiving mechanical ventilation for RDS [39, 40]. In a cohort of 26 newborns with birth weight <1250 g who were randomized to receive placebo, low dose, or high dose rhSOD within 30 minutes after the first surfactant, SOD levels in serum, tracheal aspirates, and urine significantly increased in the treatment group with respect to the placebo group [39]. The effect of the treatment was dose-dependent, with peak values of serum SOD at 6 hours, and return to basal value at 4 days for the high dose group in comparison with 12 hours and 3 days, respectively, for the low dose group. Moreover, tracheal aspirates of SOD-treated newborns showed lower levels of markers of acute lung injury, as neutrophil chemotactic activity and albumin concentration, suggesting that endotracheal rhSOD administration would exert a protective role in the acute phase of RDS. Because a single administration induced an increase in SOD levels lasting for a period of time likely inadequate to affect the risk of BPD, a regimen of repeated endotracheal administration of SOD was subsequently studied [40]. A cohort of 36 preterm newborn with birth weight <1300 g was randomized to receive placebo, low or high dose of endotracheal rhSOD within 2 hours of surfactant treatment and every 48 hours as long as mechanical ventilation was necessary, up to 7 doses. This strategy induced a 4- to 7-fold increase of SOD activity, in serum, urine, and tracheal aspirates which persisted over the 14-day study period. Despite a more prolonged anti-inflammatory effect obtained with repeated doses than with a single dose regimen, no differences in the rate of BPD were detected [40]. A larger trial including 302 preterm infants with birth weight <1200 g who received rhSOD or placebo with similar administration schedule showed lower occurrence of wheezing episodes requiring bronchodilators or corticosteroids during the first year of life in rhSOD-treated newborns [42], suggesting that very early reduction of oxidative lung injury may impact on long-term respiratory outcomes. Moreover, the subgroup of rhSOD-treated infants born before 27 weeks of gestational age experienced the more evident beneficial effect, as they presented a 55% reduction in wheezing episodes, a 55% decrease in emergency department accesses, and a 44% reduction in hospitalization when compared to the group of gestational age matched untreated infants [42]. However, no difference in the rate of BPD occurrence was detected [42]. RhSOD was well tolerated in all treated newborns [39–41]. The failure of endotracheal rhSOD treatment to reduce the rate of BPD suggests that a combination treatment with anti-inflammatory agents might be considered and investigated in the preterm newborn in order to assess possible additive effects.

Further studies on the possibility to increase the lung content of AOEs in models of preterm newborns with hyperoxia-induced lung injury were performed with novel genetic techniques as lung targeted AOEs overexpression or AOEs containing-plasmid transfection. Particularly, lung targeted human SOD3 overexpression was studied in the newborn mice model of hyperoxia-induced lung injury [48] basing on the demonstration that SOD3 overexpression reduced lung adhesion molecules and inflammatory cytokines in adult models under hyperoxic conditions [54]. After 7 days of hyperoxia transgenic mice overexpressing SOD3 had lower inflammatory markers with respect to wild-type animals, as demonstrated by lower neutrophils in tracheal aspirates and reduced myeloperoxidase activity in the lung tissue and also showed lower content of oxidized GSH in lung homogenates. Moreover, after 21 days of hyperoxia transgenic animals showed significant preservation of the alveolar surface area and of the lung volume density in comparison with wild-type animals, suggesting a beneficial role of SOD3 overexpression during the crucial phases of lung development and rearrangement after preterm birth. Theoretically, the beneficial effects of SOD3 overexpression on lung histopathology may be due not only to the direct effect of ROS scavenging but also to the production of a discrete amount of hydrogen peroxide, which is known to be involved in tissue development and remodeling, as it takes part in apoptosis and cell proliferation [48].

The protective effects of SOD3 on postnatal lung development were further assessed in preterm animal models with lung targeted overexpression of human SOD3 during the transition from the saccular to the alveolar stage [49]. While hyperoxic challenge significantly affected alveolar development in wild-type animals, transgenic mice showed preserved proliferation of alveolar and bronchiolar epithelial cells and also presented less DNA damage and preservation of specific apical protein expression on alveolar type I cells [49].

In the hyperoxia-induced lung injury, alveolar macrophages are involved in the trigger of inflammatory response through the secretion of proinflammatory mediators, as IL-1beta and TNF-alpha, which are responsible for the recruitment of further inflammatory cells, activating in turn inflammatory and oxidative cascades [3, 13]. Therefore SOD3 overexpression, which inhibits neutrophils influx into the lung, was recently studied in addition to Antileukinate, a potent suppressor of neutrophils chemotaxis, in a mice neonatal model of hyperoxia-induced lung injury [51]. Transgenic mice with lung targeted overexpression of human SOD3 receiving Antileukinate showed lower content of neutrophils in the lung and of myeloperoxidase in the bronchoalveolar lavage as well as lower levels of OS markers and increased alveolar density in comparison with wild-type treated and transgenic untreated animals, suggesting a synergistic protection of SOD3 overexpression and Antileukinate on lung injury [51].

Finally, a plasmid containing human SOD3 was administered via aerosol to animal models under hyperoxic conditions and induced a significant increase in lung tissue of NO bioavailability and cGMP accumulation [50], which is of major importance to prevent further perfusion/ventilation mismatch in RDS patients. Moreover, SOD3-treated animals also showed lower lung content of NF-kB, suggesting a possible protective role of SOD3 on the downstream inflammatory pathways [50].

### 2.3. Genomics and Antioxidant Treatments

Individual genetic characteristics may condition to a considerable extent the onset and the magnitude of the inflammatory and immune responses triggered by specific stimuli in the fetus and in the preterm newborn [55]. Particularly, a recent model of the pathogenesis of the complications of prematurity provides that a hyperresponsive phenotype activating excessive inflammatory cytokines and ROS production increases the risk of OS-related complications [55]. Among other factors, unfavorable genetic background could include specific polymorphisms of inflammatory mediators and antioxidant enzymes [56, 57]. A single nucleotide polymorphism (SNP) is a substitution or a deletion of a single nucleotide from the original DNA sequence which occurs every 100–300 base pairs and in at least 1% of the population [58]. Since BPD is characterized by a variable association of alveolar and vascular developmental arrest, inflammatory changes and oxidative damage to the developing lung [2, 3] SNPs of genes involved in such pathways were the most extensively investigated. Particularly, SNPs which influence the risk of BPD in preterm newborns were identified in genes of IL-6 [59], INF-gamma [60], Toll-like receptors family and related proteins [61], molecules regulating inflammatory cells recruitment into the lung, as L-selectin [62] and macrophage migration inhibitory factor [63], and factors regulating extracellular matrix remodeling during lung development and repair, as matrix metalloproteinases [64]. On the contrary, SNPs of TNF-alpha do not seem to affect the probability of developing BPD [65].

SNPs of AOEs have been poorly investigated so far, but the assessment of SNPs leading to reduced amount or activity of AOEs with adverse effects on the respiratory outcomes could be of major importance in the clinical perspective of AOEs replacement therapy or treatment with drugs, as MT, capable of AOEs induction. The observation of SNPs with deleterious effects on AOEs activity may pose the basis for specifically targeted therapeutic interventions. The SNP Val105Ile in the subclass pi of Glutathione-S-transferase, encoding for a low-activity variant of the enzyme, was associated with increased risk of BPD in a small population of preterm newborns [66]. We recently studied 10 SNPs of SOD1, SOD2, SOD3, and CAT in a cohort of 152 preterm newborns ≤28 weeks of gestational age and we demonstrated that SOD3 SNP rs2536512, resulting in reduced enzymatic activity, showed a trend towards a protective association with BPD [56, 57]. In the same population haplotype reconstruction analysis, which investigates possible additive effects of more SNPs, demonstrated that the risk of RDS requiring mechanical ventilation was reduced by specific haplotypes of SOD1, SOD2, SOD3, while the risk of BPD was increased by a specific haplotype of SOD2, and it was increased by a single haplotype in SOD3 [56, 57]. Although data on SNPs effects on proteomics are definitely incomplete, it appears reasonable to hypothesize that unfavorable haplotypes are associated with impaired antioxidant activity, resulting in increased oxidative damage to the preterm lung.

The demonstration of the association of specific SNPs of AOEs with adverse respiratory outcomes and the clarification of the effect of the SNPs on the activity of the resultant protein, which is partly unknown as reviewed elsewhere [43], may theoretically pose the basis for individually targeted AOEs supplementation. Moreover, also MT treatment could be speculatively expected to exert beneficial effects in newborns with SNPs leading to impaired activity of AOEs. The study of MT treatment may be of particular interest in this subset of newborns in order to assess whether MT could compensate for the genetically determined antioxidant deficit. Speculatively, the early antioxidant effects of MT treatment observed in newborns may occur as a consequence of MT anti-inflammatory and scavenging activities while the MT-induced overexpression of AOEs may be a major point in determining later effects [37], particularly in case of multiple dose treatments. However, neither AOEs activity nor the production of mRNA for AOEs have been investigated in newborns following MT treatment.

## 3. Surfactant, an Antioxidant Treatment?

Endotracheal surfactant administration was definitely among the main contributors to the improvement of the outcome of preterm birth, as it was proved to significantly reduce mortality, the need for mechanical ventilation, and the occurrence of air leaks in preterm newborn. However, there is increasing evidence that surfactant not only reduces alveolar surface tension and improves lung mechanics [67], but also possesses a complex of anti-inflammatory and antioxidant properties [68–70], which is only partially known and which may possibly contribute to the beneficial effects in RDS.

In fact, exogenous surfactant contains definite amounts of enzymatic and nonenzymatic antioxidant molecules, as summarized in Table 3, which would reduce alveolar ROS accumulation and prevent local activation of OS pathways and ROS diffusion; moreover, exogenous surfactant also replaces endogenous surfactant, which is partially impaired by ROS [70]. Indeed, lower levels of OS markers were found in tracheal aspirates of surfactant treated preterm neonates with respect to untreated preterm controls [71].

Natural calf lung surfactant contains measurable amounts of SOD and CAT and demonstrated scavenging activity against H_2_O_2_ [70] and endotracheal administration of surfactant induced a significant increase of SOD content in alveolar type II cells, demonstrating the occurrence of enzyme uptake via liposome during the surfactant recycle process [70]. It was recently assessed that 4 natural surfactants, Poractant, Beractant, Calfactant, and Bovactant, contain definite amounts of SOD and CAT [68]. Particularly, Poractant provided the major amount of SOD per recommended dose and showed the highest scavenger activity when incubated with H_2_O_2_, while Calfactant presented the major amount of CAT per dose [68]. However, the role of AOEs of surfactant in vivo still remains to be established.

Surfactant was also studied as a possible vehicle for AOEs to the intracellular space of the alveolar epithelial cells. Lung epithelial cells incubated with Beractant plus SOD and CAT showed higher SOD and CAT activity relative to cells incubated with enzymes or Beractant alone and these data were also confirmed in vivo [72]. Liposome encapsulation of SOD and CAT was also studied to enhance intracellular delivery of AOEs and such method was proved effective in increasing AOEs activity in the alveolar cells of preterm animal models of hyperoxia-induced lung injury [73]. In vitro, the addition of SOD to natural surfactants resulted in increased scavenger activities [68] and the addition of both SOD and CAT led to a higher increase in comparison with SOD or CAT alone [68].

Natural surfactants also contain antioxidants not possessing direct catalytic activity* per se*, as plasmalogens and polyunsaturated phospholipids (PUPLs) [74]. Among natural surfactants, Poractant showed the highest concentration of both plasmalogen and PUPLs [67, 74]. Plasmalogens were recently demonstrated to reduce lipid peroxidation within cell membranes [75] and also to exert antioxidant functions in low density lipoproteins [76]. Moreover, plasmalogens were shown to cooperate with cholesterol, surfactant protein B (SP-B) and SP-C for the assembly of surfactant reservoir and for the proper disposition of dipalmitoylphosphatidylcholine, and plasmalogens addiction to surfactant in experimental models led to further reduction of surface tension in comparison to surfactant alone [67, 74]. Instead, PUPLs are hypothesized to work mainly as substrates for lipid peroxidation in lung surfactant [74]. Interestingly, in tracheal aspirates collected at birth in preterm infants, the concentration of both plasmalogens and PUPLs resulted significantly higher in newborns who subsequently developed BPD in comparison to non-BPD controls [77].

Finally, thanks to anti-inflammatory and immune-modulating properties, surfactant indirectly contributes to lower lung OS. SP-A and SP-D not only serve as collectins, favoring the clearance of alveolar pathogens [78, 79], but they also suppress the proinflammatory pathway mediated by NF-kB [80], inhibit cytokines secretion, and modulate lymphocytes proliferation [62]. The role of SP-D may be of particular importance, as lower levels of SP-D were detected in tracheal aspirates of preterm newborns who subsequently developed BPD relative to age-matched non-BPD controls [81]. Furthermore, SP-D modified by reactive nitrogen species was proved to lose aggregating activity [79], and a genetic variant of SP-D was recently found to be overrepresented in RDS patients, relative to non RDS controls [82].

## 4. Conclusions

OS stress plays a key role in the pathogenesis of respiratory disease of the preterm newborn, as a consequence of immature and inadequate antioxidant capacities in the presence of multiple oxidative stimuli, resulting from preterm birth, intensive care management, and inflammatory complications.

Different antioxidant strategies are under development in order to prevent and treat respiratory diseases of prematurity, particularly with the scope of BPD prevention, since current preventive strategies and medical treatment are definitely suboptimal. MT has been poorly investigated for this indication as far as now, but encouraging results were obtained in preterm newborns and animal models, indicating MT-induced suppression of OS pathways and upregulation of AOEs. These data suggest that MT may be considered as a valuable candidate for future researches in this field. Recent evidence also suggests potential protective effects of AOEs supplementation or overexpression against OS-induced lung injury. However, only a minority of available data were obtained from clinical settings; therefore larger clinical trials are mandatory in order to clarify therapeutic potentials of such strategies.

Genetic factors predisposing to OS-related complications of prematurity are poorly known. SNPs of AOEs were recently investigated in this population and specific associations of SNPs of SOD and CAT were demonstrated to affect the risk of BPD. A more extensive assessment of SNPs involved in the pathogenesis of newborn lung diseases may be particularly helpful to identify specific populations with particularly high risk of adverse outcome, who may maximally benefit from antioxidant therapies and the clarification of the effects of SNPs on proteomics may pose the basis for an individually-targeted AOEs supplementation.

Finally, surfactant replacement, a routine treatment for RDS patients, could exert lung antioxidant effects, as AOEs and nonenzymatic antioxidants are naturally present in animal-derived surfactants. However, their role in vivo remains to be clarified.

## Figures and Tables

**Table 1:** Exogenous administration of rhSOD to preterm newborns with RDS.

Eligible population	Administration details	Outcomes	Reference
Preterm infants BW 750–1250 g <24 hrs of life (*n* = 26)	Route: ET Dose: 0.5 mg/Kg or 5 mg/Kg or saline, within 30 mins of surfactant, single dose	Reduced neutrophils chemotactic activity and albumin in tracheal aspirates in the high dose treatment group versus low dose or saline.	[39]

Preterm infants BW 700–1300 g <24 hrs of life (*n* = 33)	Route: ET Dose: 2.5 mg/Kg or 5 mg/Kg or saline, within 2 hrs of surfactant, up to 7 doses	Reduced neutrophils chemotactic activity and albumin in tracheal aspirates in the treatment groups versus saline. No difference in BPD.	[40]

Long-term follow up of preterm infants enrolled in [39, 40] (*n* = 46)	As in [39, 40]	No difference in neurodevelopmental disorder or chronic respiratory disorders at median of 28 months of corrected age.	[41]

Preterm infants BW 600–1200 g <24 hrs of life (*n* = 302)	Route: ET Dose: 5 mg/Kg or saline, within 0.5–4 hrs of surfactant, repeated every 48 hrs until 28 days	No difference in BPD rate, days of mechanical ventilation, and oxygen requirement. Reduced episodes of wheezing requiring bronchodilators or steroids in the treatment groups versus saline at 1-year follow-up.	[42]

BW: birth weight; ET: endotracheal.

**Table 2:** Exogenous administration of SOD/CAT or SOD overexpression in animal models of hyperoxic lung injury (modified from [43]).

Models	Administration or technique details	Outcomes	Reference
Rats, hyperoxic	Formulation: liposome-encapsulated bovine SOD and CAT Route: IV Timing: before and during exposure	Increased survival rate and reduced pleural effusion in the treatment group versus saline	[44]

Rats, hyperoxic	Formulation: liposome-encapsulated bovine SOD and CAT Route: ET Timing: before exposure	Increased survival rate and preserved lung histology in the treatment group versus controls.	[45]

Piglets, hyperventilated and hyperoxic	Formulation: rhSOD Route: ET Timing: at start of exposure	Reduced neutrophils chemotactic activity, total cell count, elastase activity, and albumin in tracheal aspirates versus controls.	[46]

Rabbits, lung tissue challenged with xanthine and xanthine oxidase	Formulation: liposome-encapsulated bovine SOD and CAT Route: ET	Preserved lung filtration coefficient in treated animals versus controls.	[47]

Transgenic mice, hyperoxic	Lung-targeted SOD3 overexpression	Reduced lung neutrophils and oxidized GSH, increased alveolar surface and lung volume density in transgenic animals versus wild-type controls.	[48]

Transgenic mice, hyperoxic	Lung-targeted SOD3 overexpression	Preserved alveolar and bronchiolar epithelium proliferation, reduced DNA damage, and preserved apical protein expression on type I cells in transgenic animals versus wild-type controls.	[49]

Rabbit, hyperoxic	Transfection with rhSOD3 containing plasmid Route: AER	Increased lung cGMP and decreased lung NF-kB expression in transfected animals versus controls.	[50]

Transgenic mice, hyperoxic	Lung-targeted SOD3 overexpression + Antileukinate for 7 days Route: IP	Reduced lung neutrophils, 8-isoprostane, and oxidized GSH and reduced myeloperoxidase activity in tracheal aspirates of treated transgenic animals versus treated wild type or untreated transgenic animals.	[51]

IV: intravenous; ET: endotracheal; IP: intraperitoneal; AER: aerosolized.

**Table 3:** Content of AOEs and nonenzymatic antioxidants of natural surfactants.

	Poractant	Beractant	Bovactant	Calfactant
Doses				
mg of PLs/Kg	200	100	100	100
mL of surfactant/Kg	2.5	4	2.2	2.86
SOD				
U/mg of PLs	0.396	0.474	0.027	0.383
U/mL of surfactant	31.7	11.9	1.21	13.4
U/dose per Kg	73.3	47.6	2.6	38.3
CAT				
nmol/min/mL of PLs	0.81	2.60	1.58	3.23
nmol/min/mL of surfactant	64.80	65.00	71.10	113.10
U/dose per Kg	149.80	260.00	157.80	323.50
Plasmalogens				
mol % of totalPLs	3.8 ± 0.1	1.5 ± 0.2	0.9 ± 0.3	n.a.
PUPLs				
mol % of totalPLs	26 ± 1	6 ± 1	11 ± 1	n.a.

PLs: phospholipids, PUPLs: polyunsaturated phospholipids, n.a.: not available.

## List of Abbreviations

AOE: Antioxidant enzyme
BPD: Bronchopulmonary dysplasia
CAT: Catalase
FiO_2_: Fractional inspired oxygen
GPx: Glutathione peroxidase
GRd: Glutathione reductase
GSH: Glutathione
GST: Glutathione-S-transferase
HIE: Hypoxic ischemic encephalopathy
MMP: Matrix metalloproteinase
MT: Melatonin
NO: Nitric oxide
OS: Oxidative stress
PUPLs: Polyunsaturated phospholipids
RDS: Respiratory distress syndrome
RhSOD: Recombinant human superoxide dismutase
ROS: Reactive oxygen species
SOD: Superoxide dismutase
SNP: Single nucleotide polymorphism
SP-A/D: Surfactant protein A/D.
